# Changes in Self-Reported Physical Activity Predict Health-Related Quality of Life Among South African Schoolchildren: Findings From the DASH Intervention Trial

**DOI:** 10.3389/fpubh.2020.492618

**Published:** 2020-09-30

**Authors:** Stefanie Gall, Cheryl Walter, Rosa du Randt, Larissa Adams, Nandi Joubert, Ivan Müller, Siphesihle Nqweniso, Uwe Pühse, Harald Seelig, Danielle Smith, Peter Steinmann, Jürg Utzinger, Markus Gerber

**Affiliations:** ^1^Department of Sport, Exercise and Health, University of Basel, Basel, Switzerland; ^2^Department of Human Movement Science, Nelson Mandela University, Port Elizabeth, South Africa; ^3^Swiss Tropical and Public Health Institute, Basel, Switzerland; ^4^University of Basel, Basel, Switzerland

**Keywords:** intervention, mental health, physical activity, psychosocial well-being, schoolchildren, South Africa

## Abstract

**Introduction:** Regular physical activity is associated with multiple health benefits for children. Evidence from cross-sectional studies suggests that physical activity is positively associated with health-related quality of life (HRQoL). The promotion of physical activity, and hence HRQoL, through a school-based intervention is therefore an important endeavor, particularly in disadvantaged areas of low- and middle-income countries, including South Africa.

**Methods:** We designed a multicomponent physical activity intervention that was implemented over a 20-week period in 2015 in eight disadvantaged primary schools of Port Elizabeth, South Africa. Overall, 758 children aged 8–13 years participated. HRQoL was measured with the 27-item KIDSCREEN questionnaire. Self-reported physical activity was assessed with a single item of the Health-Behavior of School-Aged Children test, and cardiorespiratory fitness with the 20-m shuttle run test. Post-intervention scores were predicted with mixed linear regression models, taking into consideration the clustered nature of the data.

**Results:** Higher baseline levels as well as increasing levels of self-reported physical activity predicted all dimensions of children's HRQoL. Baseline levels and increases in cardiorespiratory fitness predicted children's self-perceived physical well-being (one of the HRQoL subscales). Participation in the multicomponent physical activity intervention did not affect children's HRQoL.

**Conclusion:** Higher and increasing self-reported physical activity predict all assessed HRQoL dimensions, which underlines that the promotion of regular physical activity among children living in disadvantaged settings is an important public health measure. Policy makers should encourage schools to create physical activity friendly environments, while schools should implement regular physical education as proposed by the school curriculum.

## Introduction

Physical activity is a cornerstone for people's health and well-being (1). With regard to children and adolescents, previous research has shown that regular physical activity is positively associated with cardiovascular health (2), stronger bones (3), better weight control (4), lower depressive symptoms (5), improved sleep (6), positive cognitive development and academic achievements (7). Additionally, physical activity is associated with more favorable overall physical and mental well-being (8). There is also evidence from cross-sectional studies that children who regularly engage in physical activities report higher health-related quality of life (HRQoL) (9–11). Although different definitions of HRQoL exist in the literature (12, 13), in the present study, HRQoL is considered as a multidimensional construct that comprises of physical, mental, emotional, social, and behavioral aspects of well-being and functioning (14). Among children, low HRQoL is a predictor of poor childhood development, including lower educational attainment (15). HRQoL is therefore an important target variable in child research and for health interventions (16, 17).

Several pathways have been proposed on how regular physical activity might impact HRQoL (8). These pathways include the release of neurotransmitters that improve mood (18), the strengthening of social ties with peers and adults (19), and healthy, sufficient sleep (6). Furthermore, physical activity has the potential to promote stress resilience (11) by decreasing the release of stress hormones when being exposed to psychosocial stress (20). Evidence also suggests that regular physical activity has a favorable impact on brain functioning (21), which in turn contributes to children's psychosocial well-being (22).

Against this background, the World Health Organization (WHO) recommends at least 60 min of moderate-to-vigorous intensity physical activity (MVPA) for children and young people (aged 5–17 years) (23). Yet, this recommendation is not met all over the world. For instance, a report comparing 38 countries from six continents found that worldwide, only four out of 10 children and youth meet this physical activity guideline (24). The situation is particularly critical in low- and middle-income countries (LMICs), including South Africa (25), where many children only have limited access to safe physical activity environments (26).

Schools are considered an appropriate platform for the promotion of physical activity, since a large number of children are reached and a considerable amount of children's daily physical activity can be acquired during school hours (27). As shown in previous research, schools can contribute to children's physical literacy through quality physical education lessons (28). It has also been shown that multi-dimensional physical activity interventions have a positive effect on children's body mass index (BMI) (29) and have the potential to positively influence children's academic achievements (30). However, equivocal results were reported in studies examining the impact of school-based physical activity interventions on HRQoL. Although many studies found that there were significant positive effects of a physical activity intervention on HRQoL (31–33), others did not detect such effects (34–36). In their meta-analysis, Wu et al. (8) identified 31 studies, in which researchers explored the relationship between physical activity, sedentary behavior, and HRQoL in the general population of children and adolescents. Of these, 21 studies were cross-sectional, whereas seven studies used a longitudinal design and three studies examined the effects of a school-based physical activity intervention. Importantly, none of the identified studies pertained to children from an African country. Among longitudinal studies, six found that higher levels of physical activity predicted higher HRQoL over time (37–42). The only study that did not find a significant relationship used a very long (22-year) follow-up period (43). Taken together, the school-based intervention trials provided mixed findings. Whereas a school-community program in Australia improved HRQoL among adolescent girls (32), no (or a very limited) impact of a daily physical education intervention was observed in Swiss primary schoolchildren (33, 34).

The empirical evidence with regard to the prospective association between physical activity and HRQoL is growing. Nevertheless, to date, research on children from African countries is completely missing. Additionally, little is known about the potential of school-based interventions (8, 44). This is particularly true for studies in resource-poor settings, where opportunities for physical activity inside and outside the school environment are lacking. Nevertheless, a few studies suggest that increased physical activity might be particularly beneficial for children living in disadvantaged settings. For instance, Crews et al. (45) found that aerobic exercise training had a positive impact on psychological well-being among Hispanic children living in low socioeconomic districts in the United States of America. To our knowledge, only two cross-sectional studies have been conducted in South Africa, investigating the relationship between schoolchildren's physical activity and HRQoL (9, 46). Van Hout et al. (46) found that those children who participated in sportive activities at least twice a week, reported better quality of life than their inactive peers. Similarly, Salvini et al. (9) showed that schoolchildren who reported that they were active on at least 6 days a week for a minimum of 60 min per day, self-reported higher HRQoL than their peers with lower physical activity levels.

In light of these findings and in view of the absence of physical education lessons in most South African schools in disadvantaged areas (47), the current paper examines whether and to what extent (i) participation in a school-based physical activity intervention vs. a control condition; (ii) baseline levels of physical activity and cardiorespiratory fitness; and (iii) changes in physical activity and cardiorespiratory fitness, predict children's HRQoL over time. Exploring the effect of physical activity on different aspects of physical, psychological, and social functioning of HRQoL among schoolchildren will help to establish an evidence-base for public health policy decision makers, and to judge whether the promotion of physical education and physically active lifestyles is worthwhile. Based on the literature reviewed above, we hypothesized that a multi-dimension physical activity intervention could have a positive effect on children's HRQoL. Given the mixed results observed in intervention studies, we further assumed that children's overall physical activity and fitness levels (as assessed at baseline), as well as positive changes in these two variables would have an even stronger impact on HRQoL.

## Methods

### Study Design

Data presented in this paper are based on a cluster-randomized controlled trial that served to evaluate the potential impact of several school-based health promotion measures to improve the health and well-being of primary schoolchildren in disadvantaged settings in the Nelson Mandela Bay district, Port Elizabeth region, South Africa. In brief, trial schools were randomized based on a computer-generated number list either to one of four interventions or to a control condition. As described previously (29, 30, 48), schools were randomly allocated to one of the following intervention combinations: (i) physical activity alone; (ii) physical activity plus health and hygiene education; (iii) physical activity plus health and hygiene education plus nutrition education; and (iv) health and hygiene education plus nutrition education. Four schools served as control group and did not receive any intervention. Table 1 provides specific information regarding the intervention combinations and the socio-demographic background of the students at baseline in each of the participating schools.

**Table 1:** Intervention measures and baseline demographics at the eight primary schools in Port Elizabeth, South Africa.

**Condition**	**School**		**Gender**				
		**Children**	**Male**	**Female**	**Age**	**BMI**	**SES**
		**N**	**N**	**N**	**M (± SD)**	**M (± SD)**	**M (± SD)**
Physical activity	1	75	38	37	9.5 (1.0)	18.1 (4.0)	8.8 (0.5)
Physical activity + health education	2	85	39	46	9.1 (0.6)	16.9 (2.5)	7.9 (2.0)
Physical activity + health education + nutrition	3	150	80	70	9.9 (0.9)	16.3 (2.1)	6.7 (2.1)
Health education + nutrition	4	71	38	33	9.7 (1.0)	18.0 (3.3)	7.5 (1.6)
Control	5	76	37	39	9.8 (0.8)	15.5 (2.0)	6.6 (2.2)
Control	6	97	47	50	9.0 (0.8)	17.2 (2.7)	8.7 (0.9)
Control	7	121	66	55	9.5 (0.9)	17.3 (3.1)	6.5 (2.8)
Control	8	83	40	43	9.2 (0.7)	17.1 (3.0)	8.2 (1.3)

The physical activity intervention component was thus carried out in three of the eight schools. It lasted for 20 (school) weeks and contained four elements: (i) two 40 min physical education lessons per week; (ii) one weekly 40 min moving-to-music lesson; (iii) regular in-class physical activity breaks; and (iv) improving school environments to promote physical activity, including the implementation of physical play structures (jungle gyms, monkey bars, and over- and under bars) and colorful floor-painted games. Prior to the intervention, workshops were held in which lessons and games were demonstrated and class management techniques were shared. The class teachers, supported by an experienced physical education coach, held the two physical education lessons. The lessons were pre-made and structured progressively, ~40 min in duration and contained four parts: (i) warm-up (5–10 min); (ii) fitness component (10–15 min); (iii) modified invasion games (10–15 min); and (iv) cool-down and stretching activities (5–10 min). The aerobic dancing-to-music lessons lasted 40 min and were conducted by student-dancers from the Nelson Mandela University after school to the entire grade (ranging from 80 to 160 learners at one time). The lessons were structured to contain a fast-paced dance to form the warm-up component, followed by an aerobic dancing-to-music sequence as the main component, and concluded with a slow-paced routine to music for the cool-down component. Baseline assessment took place between January and March 2015, whereas the post-intervention assessment was carried out between May and June 2016.

### Ethics Statement

The study was approved by the ethics committee of Northwestern and Central Switzerland (EKNZ; reference no. 2014-179, approval date: 17 June 2014), the Nelson Mandela University (NMU) Ethics Committee (study number H14-HEA-HMS002, approval date: 4 July 2014) and the ethics review boards of the Eastern Cape Department of Education (approval date: 3 August 2014), and the Eastern Cape Department of Health (approval date: 7 November 2014). The study is registered at ISRCTN registry under controlled-trials.com (unique identifier: ISRCTN68411960, registration date: 1 October 2014). Prior to beginning the data assessment, written informed consent was obtained from the parents/legal guardians of children, while children assented orally. All procedures were in line with the ethical principles described in the Declaration of Helsinki.

### Participants and Procedures

Eight primary schools participated in the Disease, Activity and Schoolchildren's Health (DASH) study. Schools were selected according to geographic location, representation of the target communities, and commitment shown by school principals to support the project activities. The detailed inclusion criteria can be found in the study protocol (48). Participation in the study was voluntary and children could withdraw at any time and without any further obligation.

The initial sample at baseline consisted of 1,009 children [508 girls, 501 boys, mean age at baseline=9.5 years, standard deviation (SD) = 0.9 years]. For the present data analyses, 190 children were excluded because they left the study between the baseline assessment and post-intervention. Moreover, 61 children were excluded due to missing baseline data in at least one of the covariates [i.e., age, gender, BMI, and socioeconomic status (SES)]. Thus, the final sample for the current analyses consisted of 758 children (373 girls, 385 boys). Hereof, 448 belonged to the control group (220 girls, 228 boys, mean age at baseline = 9.4 years, SD = 0.9 years), whereas 310 received the physical activity intervention component (153 girls, 157 boys, mean age at baseline = 9.6 years, SD = 0.9 years). We performed a series of univariate analyses of variance (ANOVAs) (for metric study variables) and χ^2^-tests (for categorical variables), in order to compare those 758 children who were included in the present analyses with those who dropped out or were excluded due to missing data. No significant differences (*p* > 0.05) existed between these two groups in any of the covariates, the predictor or outcome variables. Moreover, included/excluded students were similarly represented in the intervention and control condition.

### Measures

The same indicators were assessed before the start, and after completion of the intervention. The data assessment was carried out class-wise during official school hours by trained research officers. SES was assessed with a nine-item self-report questionnaire about housing characteristics, ownership of durable assets (e.g., washing machine), and household-level living standards. Scores of the SES index ranged from one to nine, with higher scores being indicative of higher family SES. Evidence for the validity of similar SES scales has been reported in previous studies (49). To assess self-perceived well-being, children completed the 27-item KIDSCREEN (50). Answers were given on a 5-point Likert scale ranging from “never” to “always.” This instrument is composed of five subscales labeled physical well-being, psychological well-being, autonomy and parent relation, peers and social support, and school environment. To obtain an overall estimate of children's HRQoL, we calculated the mean across all five KIDSCREEN dimensions. Additionally, we calculated the 10-item overall HRQoL index, as suggested in the KIDSCREEN manual (51). The reliability and validity of the KIDSCREEN has been documented previously (52). Following the official scoring guidelines, for each dimension, raw scores were transformed into Rasch person parameter estimates using the available IBM SPSS statistics software version 25 (IBM Corp; Armonk, NY, United States of America) syntax for each dimension. The calculated Rasch scores had a scale mean of 50 and a SD of 10 (50), with higher scores reflecting better well-being and HRQoL. To assess physical activity behavior, the children were asked to answer a single-item question taken from the Health-Behavior of School-Aged Children (HBSC) survey (53): “*Over the past 7 days (1 week), on how many days were you physically active for a total of at least 60 min (1 h) a day?”* Answering options ranged from 0 to 7 days. Previous studies showed that this question has acceptable validity when compared with physical activity assessed by accelerometers (54, 55); it has previously been used in studies measuring physical activity and HRQoL (55). Finally, to assess children's cardiorespiratory fitness, the 20-m shuttle run test was employed (56). A 20 m flat course was laid out and marked with 10–15 color coded cones. Children ran back and forth according to a sound signal on the premeasured running court and they were accompanied by a trained researcher officer. The pre-recorded sound signal started at a speed of 8.5 km/h and steadily increase by 0.5 km/h every minute. If a child was unable to cross the marked 2 m line at the moment of the sound signal for two consecutive intervals, the child was asked to stop and only the fully completed laps were noted.

All children underwent a clinical examination by a registered medical nurse to identify any health problems. Children who did not pass the health examination were excluded from the maximal exercise test. The 20-m shuttle run test has a moderate-to-high mean criterion-related validity for estimating cardiorespiratory fitness (57).

### Statistical Analysis

Data were double entered and validated using EpiData version 3.1 (EpiData Association; Odense, Denmark). Statistical analyses were performed with IBM SPSS statistics version 25 for Windows (IBM Corp.; Armonk, NY, United States of America) and STATA version 13.0 (STATA Corp.; College Station, TX, United States of America). For metric study variables, univariate ANOVAs were utilized to test differences between the intervention and control group at baseline and at post-intervention, whereas χ^2^ tests were employed for examining differences in categorical variables. To examine whether intervention allocation (physical activity vs. control condition), baseline levels of physical activity and cardiorespiratory fitness, and changes in physical activity and cardiorespiratory fitness from baseline to post-intervention predicted HRQoL, a series of mixed linear regression analyses were performed, with random intercepts for school classes, in order to adjust for cluster effects. These analyses were carried out with the multilevel mixed effects linear regression procedure (covariance structure = independent) in STATA. Separate analyses were carried out for the five KIDSCREEN subscales and the two overall HRQoL indices. Before testing the effect of the condition (physical activity vs. control), all regression analyses were controlled for children's age, gender, BMI, SES, and baseline KIDSCREEN scores. Change scores were calculated by subtracting baseline scores from post-intervention scores. For all regression analyses, we display the unstandardized Beta coefficients and the 95% confidence intervals (CIs). Statistical significance was set at *p* < 0.05 across all analyses. Based on a detailed missing data inspection, we found no evidence of systematic missing data pattern. Hence, we decided not to impute missing data, and to perform the regression analyses with data of children who had complete data records.

## Results

### Descriptive Statistics

Table 2 provides an overview of the descriptive statistics and group differences at baseline and at post-intervention assessment, for all study variables. Our study sample consisted of 758 children from eight primary schools in Port Elizabeth. At baseline, children were aged between 8 and 12 years, 50.8% were boys.

**Table 2:** Means (M) and standard deviations (SD) at baseline and post-intervention in wellbeing, physical activity and cardiorespiratory fitness, and differences between intervention and control group.

	**Control group (*n* = 448)**		**Intervention group (*n* = 310)**		**ANOVAs**		
**Baseline**	**M (± SD)**		**M (± SD)**		**F**		**η2**
Age	9.4 (0.9)		9.6 (0.9)		7.0**		0.009
BMI	17.1 (3.0)		16.9 (2.9)		0.6		0.001
BMI *z*-score	0 (1.2)		−0.1 (1.2)		1.3		0.002
Socioeconomic status^a^	7.5 (2.1)		7.5 (2.0)		0.3		0.000
Self-reported physical activity	3.1 (2.4)		4.0 (2.5)		29.1***		0.037
Shuttle run laps	36.5 (17.4)		35.0 (17.0)		1.3		0.002
Cardiorespiratory fitness (VO_2_max)	49.4 (4.3)		48.8 (4.2)		3.7		0.005
Physical wellbeing (KIDSCREEN 27)	50.0 (13.6)		51.2 (13.0)		1.5		0.002
Psychological wellbeing (KIDSCREEN 27)	37.3 (8.3)		39.5 (8.8)		11.6**		0.015
Autonomy and parent relations (KIDSCREEN 27)	49.9 (12.6)		48.8 (12.2)		1.2		0.002
Social support and peers (KIDSCREEN 27)	48.0 (11.8)		49.9 (11.8)		4.7*		0.006
School environment (KIDSCREEN 27)	53.8 (12.1)		57.4 (12.6)		16.1***		0.021
Overall HRQoL: Mean across dimensions (KIDSCREEN 27)	47.8 (8.8)		49.4 (8.6)		5.9**		0.008
Overall HRQoL: 10-item overall index (KIDSCREEN 10)	50.1 (12.8)		51.9 (15.2)		3.0		0.004
**Post-intervention**	**M (±** **SD)**		**M (±** **SD)**		**F**		***η*****2**
Age	10.6 (0.9)		10.9 (0.9)		13.3**		0.012
BMI	18.1 (3.6)		17.8 (3.6)		1.9		0.003
BMI *z*-score	0.1 (1.3)		−0.1 (1.3)		4.4*		0.006
Self-reported physical activity	4.7 (2.3)		4.3 (2.2)		4.3*		0.006
Shuttle run laps	35.1 (18.6)		34.8 (21.1)		0.0		0.000
Cardiorespiratory fitness (VO_2_max)	47.0 (4.7)		46.5 (4.5)		2.4		0.003
Physical wellbeing (KIDSCREEN 27)	46.7 (9.7)		46.5 (9.1)		0.1		0.000
Psychological wellbeing (KIDSCREEN 27)	48.1 (11.8)		46.3 (11.1)		4.6*		0.006
Autonomy and parent relations (KIDSCREEN 27)	46.6 (10.5)		46.4 (9.6)		0.1		0.000
Social support and peers (KIDSCREEN 27)	48.0 (11.2)		46.6 (10.4)		2.9		0.004
School environment (KIDSCREEN 27)	53.3 (12.2)		51.6 (11.6)		3.8		0.005
Overall HRQoL: Mean across dimensions (KIDSCREEN 27)	48.6 (8.2)		47.5 (7.2)		3.4		0.004
Overall HRQoL: 10-item overall index (KIDSCREEN 10)	47.7 (11.1)		46.1 (9.5)		3.9*		0.005
	**Control group (*****n*** **=** **448)**		**Intervention group (*****n*** **=** **310)**		***χ***^**2**^ **tests**		
**Categorical variables**	***N***	**%**	***N***	**%**	***χ***^**2**^	**ϕ**	
Gender					0.0	0.002	
Girls	220	49.1	153	49.4			
Boys	228	50.9	157	50.6			

*HRQoL = Health-related quality of life*.

a *Socioeconomic status was only assessed at baseline*.

*p < 0.05, **p < 0.01, ***p < 0.001.

### Differences Between Intervention and Control Group at Baseline and Post-intervention

Table 2 also shows the descriptive statistics separately for children assigned to the intervention and control group. Based on univariate ANOVAs, the intervention and control group significantly differed with regard to age, self-reported physical activity, and three of the KIDSCREEN subscales (psychological well-being, social support and peers, and school environment) as well as the overall HRQoL (mean across dimensions). The intervention group was slightly older, had higher self-reported physical activity, higher psychological well-being, higher scores in the domain social support and peers, school environment, and in the overall mean index compared to the control group. At post-intervention, significant group differences disappeared except for the difference in age, self-reported physical activity, psychological well-being, and overall HRQoL (10 item).

### Prediction of HRQoL

#### Prediction of Post-intervention Scores

Table 3 summarizes the results of the mixed linear regression analyses, highlighting variables that acted as predictors of children's HRQoL. In Model 1, the dependent variable was the HRQoL scores at post-intervention, and analyses were computed separately for the five KIDSCREEN subscales and the two overall HRQoL indices.

**Table 3:** Multiple mixed linear regression analyses to predict post-intervention scores in children's health-related quality of life.

**Prediction of KIDSCREEN post-intervention scores (*****N*** **=** **758)**																					
Model 1^*^	**Physical well-being**			**Psychological well-being**			**Autonomy and parent relations**			**Social support and peers**			**School environment**			**Overall HRQoL: Mean across dimensions**			**Overall HRQoL: 10-item index**		
	**B**^**a**^	**Estimate**^**b**^ **(95% CI)**	***p*****-value**^**c**^	**B**^**a**^	**Estimate**^**b**^ **(95% CI)**	***p*****-value**^**c**^	**B**^**a**^	**Estimate**^**b**^ **(95% CI)**	***p*****-value**^**c**^	**B**^**a**^	**Estimate**^**b**^ **(95% CI)**	***p*****-value**^**c**^	**B**^**a**^	**Estimate**^**b**^ **(95% CI)**	***p*****-value**^**c**^	**B**^**a**^	**Estimate**^**b**^ **(95% CI)**	***p*****-value**^**c**^	**B**^**a**^	**Estimate**^**b**^ **(95% CI)**	***p*****-value**^**c**^
Age	−0.36	−1.14–0.41	0.361	−1.99	−2.95 – −1.03	0.000***	−0.17	−1–0.65	0.678	−0.78	−1.67–0.11	0.087	−0.77	−1.74–0.2	0.119	−0.75	−1.37–−0.13	0.018^*^	−1.20	−2.04 – −0.36	0.005**
Gender (1 = male, 2 = female)	1.88	0.37–3.38	0.014^*^	1.24	−0.61–3.1	0.190	1.29	−0.31–2.9	0.114	−0.21	−1.96–1.54	0.815	4.77	2.86–6.68	0.000***	1.76	0.55–2.96	0.004**	1.73	0.1–3.36	0.037^*^
BMI *z*-scores	0.00	−0.02–0.01	0.697	0.02	−0.01–0.04	0.161	0.00	−0.02–0.02	0.847	0.01	−0.01–0.03	0.261	0.00	−0.02–0.02	0.824	0.01	−0.01–0.02	0.444	0.00	−0.02–0.02	0.866
Socioeconomic status	0.41	0.08–0.74	0.016^*^	0.24	−0.17–0.65	0.259	0.39	0.03–0.74	0.032^*^	0.31	−0.07–0.69	0.112	0.64	0.22–1.05	0.002**	0.48	0.22–0.75	0.000***	0.29	−0.07–0.65	0.110
Baseline score of KIDSCREEN dimension	0.09	0.04–0.15	0.000***	0.00	−0.1–0.1	0.966	0.17	0.1–0.23	0.000***	0.18	0.12–0.25	0.000***	0.15	0.08–0.22	0.000***	0.14	0.09–0.18	0.000***	0.20	0.15–0.26	0.000***
Condition (1 = control, 2 = intervention)	−0.24	−2.62–2.14	0.843	−1.94	−5–1.13	0.215	−0.14	−2.77–2.5	0.918	−1.88	−3.97–0.22	0.079	−1.76	−4.26–0.75	0.169	−1.20	−3.25–0.84	0.250	−1.78	−4.67–1.11	0.227
Baseline physical activity	0.50	0.09–0.91	0.016^*^	0.69	0.18–1.19	0.008**	0.59	0.15–1.03	0.008**	0.88	0.41–1.35	0.000***	0.57	0.05–1.08	0.030^*^	0.57	0.24–0.9	0.001**	0.25	−0.2–0.69	0.278
Change in physical activity^d^	0.45	0.16–0.74	0.002**	0.43	0.07–0.78	0.019^*^	0.45	0.14–0.75	0.004**	0.62	0.29–0.96	0.000***	0.64	0.28–1	0.001**	0.49	0.26–0.72	0.000***	0.26	−0.06–0.57	0.107
Baseline cardiorespiratory fitness	0.32	0.13–0.5	0.001**	0.13	−0.1–0.36	0.281	−0.04	−0.24–0.16	0.720	−0.12	−0.34–0.09	0.264	0.02	−0.21–0.26	0.847	0.06	−0.09–0.21	0.427	0.05	−0.15–0.25	0.641
Change in cardiorespiratory fitness^d^	0.26	0.08–0.44	0.005**	0.07	−0.16–0.29	0.550	−0.15	−0.34–0.04	0.132	−0.06	−0.27–0.15	0.580	−0.03	−0.26–0.2	0.797	0.01	−0.13–0.16	0.851	0.06	−0.14–0.25	0.580

* *In the mixed linear regression models, cases were excluded listwise from the analysis if they had missing data in one or several of the covariates. Thus all mixed linear regression analyses were based on data of children with complete data records across all variables: N = 758*.

a *B represents the estimate of the beta coefficient*.

b *Adjusted estimates of mean change in the respective outcome from baseline to post-intervention: Unstandardized Beta coefficients, 95% confidence interval, and p-value*.

c *All p-values are calculated using mixed linear regression, adjusting for clustering of school classes*.

d *To obtain change scores for physical activity and cardiorespiratory fitness, baseline scores were subtracted from post-intervention scores. Thus, higher change scores reflect stronger increases in physical activity and cardiorespiratory fitness*.

e *To obtain change scores for the KIDSCREEN overall index and subdimensions, baseline scores were subtracted from post-intervention scores. Thus, higher change scores reflect stronger increases in self-perceived wellbeing*.

*p < 0.05, **p < 0.01, ***p < 0.001.

With regard to the covariates, higher age was associated with lower overall HRQoL (mean across dimensions and 10-item index) and lower scores for psychological well-being at post-intervention. Girls reported better physical well-being at post-intervention, rated the school environment more positively, and reported better overall HRQoL (mean across dimensions and 10-item index). Students with higher SES reported higher scores for physical well-being, autonomy and parent relations, had a more positive perception of the school environment, and scored higher in the overall HRQoL (mean across dimensions). Except for psychological well-being, baseline scores in HRQoL significantly predicted the respective KIDSCREEN scales at post-intervention.

Condition (physical activity intervention vs. control condition) was not associated with the outcomes. Baseline levels and change of physical activity predicted all KIDSCREEN subscales (physical well-being, psychological well-being, autonomy and parent relations, social support and peers, and school environment) as well as the overall HRQoL (mean across dimensions). The positive association between the predictor and outcomes indicates that higher baseline physical activity levels predicted better HRQoL scores at post-intervention. The findings also show that increases in self-reported physical activity were associated with better HRQoL at post-intervention. Whereas, this pattern was found consistently across all KIDSCREEN subscales, no significant association was observed for the overall 10-item HRQoL index. Moreover, baseline levels of, and improvements in, cardiorespiratory fitness predicted one domain, namely better physical well-being at post-intervention.

## Discussion

The key findings of the present study highlight that assignment to the intervention or control condition did not affect the HRQoL among schoolchildren from the Port Elizabeth area in South Africa. Importantly, however, our analyses revealed that baseline levels and levels of self-reported physical activity positively (and consistently across all investigated dimensions) predicted children's HRQoL perceptions. Additionally, baseline levels and increases in cardiorespiratory fitness made a significant contribution to the prediction of children's self-perceived physical well-being.

The observation that higher self-reported physical activity levels result in higher HRQoL in our study population is important and in line with previous research. This finding might be attributed to several factors. Gopinath et al. (40) found that physically active adolescents reported higher HRQoL over a 5-year period and argued that participation in sports or games has a positive effect on the development of social reinforcement and social functioning. Physical activity contributes to the feeling of being socially accepted and popular, which are important concepts of self-perceived well-being (10). Higher levels of physical activity might be further associated with better quality of sleep, which in turn has a positive effect on children's behavioral and emotional health (6). Studies also show that children who are physically active can better cope with stress (11) and have an enhanced physical self-concept (58). Previous research has also shown that children who engage in regular physical activity report fewer depressive symptoms (5). Omorou et al. (37) found a cumulative and bidirectional association between physical activity and HRQoL for adolescents in France over a 2-year period. The authors concluded that physical activity and sedentary behavior are important components in improving adolescents' well-being as well as preventing non-communicable diseases. Vella et al. (39) found a protective effect of sport participation on HRQoL in children aged 8–10 years. They found that the maintenance of sport participation resulted in elevated HRQoL. Based on a systematic review, Lubans et al. (22) concluded that it is still unclear what kind of neurobiological and behavioral mechanism might be at play when it comes to the effects of physical activity on HRQoL. Yet, they emphasized that participation in physical activity can improve physical self-perceptions and enhance self-esteem in young people (59).

Contrary to our working hypothesis, baseline levels of, and improvements in, cardiorespiratory fitness predicted only one HRQoL domain, namely physical well-being. This observation might be explained as follows. First, similar to our study, Morales et al. (60) observed that in comparison to other HRQoL domains, there is a particularly close relationship between cardiorespiratory fitness and physical well-being. This is not unexpected because low cardiorespiratory fitness is an independent marker of cardiovascular risk (61), and thus a physical health marker. Second, even though cardiorespiratory fitness is seen as a proxy for physical activity, it is depending on genetic factors, whereas physical activity is a behavioral component (62). Third, in a study with Swedish adults, Lindwall et al. (63) observed that self-reported physical activity is more closely associated with mental health outcomes than objectively assessed cardiorespiratory fitness. The authors argued that psychological processes, such as perceived control over one's health and body, might play a more important role than improved cardiovascular change. Fourth, both physical activity and HRQoL are self-reported and may therefore suffer from reporting bias and share common method variance (64). Nevertheless, it is important to mention that numerous researchers found positive associations between cardiorespiratory fitness and HRQoL (65). For instance, Andersen et al. (66) reported that cardiorespiratory fitness is positively associated with higher scores on all five KIDSCREEN-27 domains in a cross-sectional analysis. Hence, cardiorespiratory fitness should be seen as an important target variable for public health interventions.

With regard to the investigated covariates, our results suggest that higher age was associated with lower overall HRQoL (10-item index, mean across dimensions) and lower psychological well-being at post-intervention. This is in line with prior research (67) and can be explained through the physical as well as social transition from childhood to adulthood. Subjective well-being can be impaired through an imbalance of hormones and new physiological processes (68). Bisegger et al. (68) argued that puberty is physically more radical for girls than for boys and this can contribute to a decreased psychological well-being. Yet, in our study sample, girls reported better physical well-being at post-intervention and rated the school environment more positively than boys. The finding that girls perceived the school environment more positively could be attributed to the fact that girls saw the school as a social place and a “sanctuary.” Our observation that girls perceived their overall HRQoL and their physical health more positively than boys is contrary to most studies with European children. We hypothesize that underlying social concepts might be different in the South African context. One study by Chen et al. (69) found that a higher BMI is linked to lower HRQoL, while Griffiths et al. (70) reported that excess weight may impact HRQoL due to low self-image and low self-confidence. In our study sample, BMI was not statistically significantly associated with HRQoL. This might be due to cultural differences, since being overweight is seen more positively in the local culture, as it indicates wealth and happiness (71). Finally, we found that children with higher SES rated the HRQoL subscale physical well-being, autonomy and parent relations, school environment, and overall HRQoL (mean across dimensions) more positively than their peers with lower SES. This finding accords well with a study carried out in seven European countries, in which children with lower SES reported lower HRQoL (72).

Our observation that a multi-dimensional physical activity intervention had no effect on children's HRQoL is in line with the equivocal findings reported in recent reviews. In their systematic review of school-based physical activity interventions, Rafferty et al. (44) evaluated 11 studies. Hereof, only three studies reported a positive effect on children's well-being. In a recent school-based physical activity trial, Resaland et al. (35) observed no significant effect of the intervention on HRQoL. However, it remained unclear whether the intervention indeed resulted in increased overall physical activity. In their review, Wu et al. (8) suggested that there is a dose-response relation between physical activity, sedentary behavior, and HRQoL, indicating that there is a linear relationship between higher physical activity levels (or less time spent being sedentary) and better HRQoL.

The lack of impact of our intervention on children's HRQoL may be due to limited exposure and intensity of our program. Our intervention lasted for 20 weeks, including two physical education and one moving-to-music lesson per week. Moreover, we observed that overall physical activity levels increased more in the control group, compared to the self-reported levels of physical activity which increased only slightly in the intervention group. Hence, it is conceivable that unrelated changes, or a participant bias in the physical activity behavior in control schools, have superimposed the effects of our intervention.

Although our study provides new insights with regard to the effect of regular physical activity on HRQoL in South African schoolchildren, our findings must be considered in light of several limitations. First, physical activity was assessed with a relatively simple single-item instrument. Of note, this item was successfully employed in large-scale studies with children and adolescents in a European context (HBSC study), in which meaningful relationships were found between physical activity and health-related outcomes (73). Second, no objective physical activity measurements were obtained, which would have required the use of accelerometers (74). Accelerometry data have the potential to differentiate between intensities of physical activity and should be considered in future research. Nevertheless, although the precision of physical activity data might have been limited due to the simplicity of our instrument, it is noteworthy that it was self-reported physical activity that performed best as predictor of children's HRQoL (and not cardiorespiratory fitness). Third, our impression was that the quality of the intervention implementation depended to some extent on the motivation and skills of the teachers. For future research, we therefore suggest to more systematically assess the quality of intervention implementation, and to consider this as a moderating factor. Fourth, we acknowledge that in our study there was only one school in each intervention program, and some students received physical activity alone, some students in combination with other intervention components (health and hygiene and/or nutrition), and some students not at all. While this can be seen as a limitation, we included random intercepts for school classes in order to adjust for cluster effects. As highlighted by Geiser (75), using a mixed model approach (and taking into consideration the hierarchical structure of clustered data) is important because (i) clustered samples violate some of the assumption of independence of observations which is made in traditional regression analyses, and (ii) in many studies, variables at different level (e.g., individual, class, and intervention condition) are relevant for the prediction of an outcome variable. Fifth, although the 20 m shuttle run test is a frequently used procedure to assess children's cardiorespiratory fitness, a recent meta-analysis has revealed that compared to children, the criterion-related validity of Léger's protocol was statistically higher for adults (*r* = 0.94, 95% CI 0.87–1.00). Yet, the protocol performed quite well in children (*r* = 0.78, 95% CI 0.72–0.85).

## Conclusion

Higher physical activity and positive change in physical activity was prospectively associated with better HRQoL in a sample of South African children attending primary schools from disadvantaged neighborhoods. In view of our findings, we suggest that South African policy makers might reflect on how children's overall physical activity can be increased and how schools can be supported to provide physical activity-friendly environments that promote intramural physical activity. Concerted efforts are required to implement high quality physical education in schools in disadvantaged settings in order to sustainably increase children's physical literacy and to promote physically active lifestyles.

## Data Availability Statement

The datasets generated for this study are available on request to the corresponding author.

## Ethics Statement

This study was approved by the ethical review board of Northwestern and Central Switzerland, the Nelson Mandela University (NMU) Human Ethics Committee, the Eastern Cape Department of Education, and the Eastern Cape Department of Health in Port Elizabeth, South Africa. The study is registered at ISRCTN registry under controlled-trials.com (unique identifier: ISRCTN68411960). Prior to beginning the data assessment, written informed consent was obtained from the parents/legal guardians of children, while children assented orally.

## Author Contributions

CW, IM, UP, JU, and MG: conceptualization. SG, HS, and MG: data curation. SG and MG: formal analysis and writing-original draft. CW, RdR, IM, UP, JU, and MG: funding acquisition. CW, IM, HS, PS, and MG: methodology. IM: project administration. IM, UP, HS, and MG: supervision. CW, RdR, LA, NJ, IM, SN, UP, HS, DS, PS, JU, and MG: writing-review and editing. All authors contributed to the article and approved the submitted version.

## Conflict of Interest

The authors declare that the research was conducted in the absence of any commercial or financial relationships that could be construed as a potential conflict of interest.

## References

[1] Ortega FB Ruiz JR Castillo MJ Sjostrom M. Physical fitness in childhood and adolescence: a powerful marker of health. Int J Obes. (2008) 32:1–11. 10.1038/sj.ijo.0803774 18043605

[2] Ekelund U Anderssen SA Froberg K Sardinha LB Andersen LB Brage S. Independent associations of physical activity and cardiorespiratory fitness with metabolic risk factors in children: the European youth heart study. Diabetologia. (2007) 50:1832–40. 10.1007/s00125-007-0762-5 17641870

[3] Gabel L Macdonald HM Nettlefold L McKay HA. Physical activity, sedentary time, and bone strength from childhood to early adulthood: a mixed longitudinal HR-pQCT study. J Bone Miner Res. (2017) 32:1525–36. 10.1002/jbmr.3115 28326606

[4] Janssen I Leblanc AG. Systematic review of the health benefits of physical activity and fitness in school-aged children and youth. Int J Behav Nutr Phys Act. (2010) 7:40. 10.1186/1479-5868-7-40 20459784 PMC2885312

[5] Korczak DJ Madigan S Colasanto M. Children's physical activity and depression: a meta-analysis. Pediatrics. (2017) 139:e20162266. 10.1542/peds.2016-2266 28314824

[6] Lang C Kalak N Brand S Holsboer-Trachsler E Pühse U Gerber M. The relationship between physical activity and sleep from mid adolescence to early adulthood. A systematic review of methodological approaches and meta-analysis. Sleep Med Rev. (2016) 28:32–45. 10.1016/j.smrv.2015.07.004 26447947

[7] Esteban-Cornejo I Tejero-Gonzalez CM Sallis JF Veiga OL. Physical activity and cognition in adolescents: a systematic review. J Sci Med Sport. (2015) 18:534–9. 10.1016/j.jsams.2014.07.007 25108657

[8] Wu XY Han LH Zhang JH Luo S Hu JW Sun K. The influence of physical activity, sedentary behavior on health-related quality of life among the general population of children and adolescents: a systematic review. PLoS ONE. (2017) 12:e0187668. 10.1371/journal.pone.0187668 29121640 PMC5679623

[9] Salvini M Gall S Müller I Walter C du Randt R Steinmann P. Physical activity and health-related quality of life among schoolchildren from disadvantaged neighbourhoods in Port Elizabeth, South Africa. Qual Life Res. (2017) 27:205–16. 10.1007/s11136-017-1707-1 28965191

[10] Breslin G Gossrau-Breen D McCay N Gilmore G McDonald L Hanna D. Physical activity, gender, weight status, and wellbeing in 9- to 11-year-old children: a cross sectional survey. J Phys Act Health. (2012) 9:394–401. 10.1123/jpah.9.3.394 21934158

[11] Gerber M Endes K Brand S Herrmann C Colledge F Donath L. In 6- to 8-year-old children, cardiorespiratory fitness moderates the relationship between severity of life events and health-related quality of life. Qual Life Res. (2016) 26:695–706. 10.1007/s11136-016-1472-6 27933428

[12] Karimi M Brazier J. Health, health-related quality of life, and quality of life: what is the difference? Pharmacoeconomics. (2016) 34:645–9. 10.1007/s40273-016-0389-9 26892973

[13] Ferrans CE Zerwic JJ Wilbur JE Larson JL. Conceptual model of health-related quality of life. J Nurs Scholarsh. (2005) 37:336–42. 10.1111/j.1547-5069.2005.00058.x 16396406

[14] Ravens-Sieberer U Erhart M Wille N Wetzel R Nickel J Bullinger M. Generic health-related quality-of-life assessment in children and adolescents: methodological considerations. Pharmacoeconomics. (2006) 24:1199–220. 10.2165/00019053-200624120-00005 17129075

[15] Patel V Flisher AJ Hetrick S McGorry P. Mental health of young people: a global public-health challenge. Lancet. (2007) 369:1302–13. 10.1016/S0140-6736(07)60368-7 17434406

[16] Ravens-Sieberer U Gosch A Erhart M von Rueden U Nickel J Kurth BM. The KIDSCREEN Questionnaires. Quality of Life Questionnaires for Children and Adolescents. Handbook. Lengerich: Pabst Science Publishers (2006).

[17] Freire T Ferreira G Health-related quality of life of adolescents: relations with positive and negative psychological dimensions. Int J Adolesc Youth. (2016) 23:11–24. 10.1080/02673843.2016.1262268

[18] Dishman RK O'Connor PJ Lessons in exercise neurobiology: the case of endorphins. Ment Health Phys Act. (2009) 2:4–9. 10.1016/j.mhpa.2009.01.002

[19] Daley AJ Copeland RJ Wright NP Roalfe A Wales JK. Exercise therapy as a treatment for psychopathologic conditions in obese and morbidly obese adolescents: a randomized, controlled trial. Pediatrics. (2006) 118:2126–34. 10.1542/peds.2006-1285 17079587

[20] Mücke M Ludyga S Colledge F Gerber M. Influence of regular physical activity and fitness on stress reactivity as measured with the trier social stress test protocol: a systematic review. Sports Med. (2018) 48:2607–22. 10.1007/s40279-018-0979-0 30159718

[21] Ludyga S Gerber M Herrmann C Brand S Pühse U. Chronic effects of exercise implemented during school-break time on neurophysiological indices of inhibitory control in adolescents. Trends Neurosci Educ. (2018) 10:1–7. 10.1016/j.tine.2017.11.00129358034

[22] Lubans D Richards J Hillman C Faulkner G Beauchamp M Nilsson M. Physical activity for cognitive and mental health in youth: a systematic review of mechanisms. Pediatrics. (2016) 138:e20161642. 10.1542/peds.2016-1642 27542849

[23] World Health Organization Global Recommendations on Physical Activity for Health. Geneva: WHO (2010). 26180873

[24] Tremblay MS Barnes JD Gonzalez SA Katzmarzyk PT Onywera VO Reilly JJ Global matrix 2.0: report card grades on the physical activity of children and youth comparing 38 countries. J Phys Act Health. (2016) 13(11 Suppl. 2):S343–66. 10.1123/jpah.2016-0594 27848745

[25] Draper CE Tomaz SA Bassett SH Burnett C Christie CJ Cozett C. Results from South Africa's 2018 report card on physical activity for children and youth. J Phys Act Health. (2018) 15:S406–8. 10.1123/jpah.2018-0517 30475151

[26] McHunu S Le Roux K Non-participation in sport by black learners with special reference to gender, grades, family income and home environment. S Afr J Res Sport Phys Educ Rec. (2010) 32:85–98. 10.4314/sajrs.v32i1.54102

[27] Meyer U Roth R Zahner L Gerber M Puder JJ Hebestreit H. Contribution of physical education to overall physical activity. Scand J Med Sci Sports. (2013) 23:600–6. 10.1111/j.1600-0838.2011.01425.x 22151355

[28] McLennan N Thompson J Quality Physical Education (QPE). Guidelines for Policy-Makers. Paris: UNESCO Publishing (2015).

[29] Müller I Schindler C Adams L Endes K Gall S Gerber M. Effect of a multidimensional physical activity intervention on body mass index, skinfolds and fitness in South African children: results from a cluster-randomised controlled trial. Int J Environ Res Public Health. (2019) 16:232. 10.3390/ijerph16020232 30650624 PMC6352127

[30] Gall S Adams L Joubert N Ludyga S Müller I Nqweniso S. Effect of a 20-week physical activity intervention on selective attention and academic performance in children living in disadvantaged neighborhoods: a cluster randomized control trial. PLoS ONE. (2018) 13:e0206908. 10.1371/journal.pone.0206908 30408073 PMC6224098

[31] Ha AS Burnett A Sum R Medic N Ng JY. Outcomes of the rope skipping 'STAR' programme for schoolchildren. J Hum Kinet. (2015) 45:233–40. 10.1515/hukin-2015-0024 25964826 PMC4415837

[32] Casey MM Harvey JT Telford A Eime RM Mooney A Payne WR. Effectiveness of a school-community linked program on physical activity levels and health-related quality of life for adolescent girls. BMC Public Health. (2014) 14:649. 10.1186/1471-2458-14-649 24966134 PMC4080584

[33] Hartmann T Zahner L Pühse U Puder JJ Kriemler S. Effects of a school-based physical activity program on physical and psychosocial quality of life in elementary school children: a cluster-randomized trial. Pediatr Exerc Sci. (2010) 22:511–22. 10.1123/pes.22.4.511 21242601

[34] Kriemler S Zahner L Schindler C Meyer U Hartmann T Hebestreit H. Effect of school based physical activity programme (KISS) on fitness and adiposity in primary schoolchildren: cluster randomised controlled trial. BMJ. (2010) 340:c785. 10.1136/bmj.c785 20179126 PMC2827713

[35] Resaland GK Aadland E Moe VF Kolotkin RL Anderssen SA Andersen JR. Effects of a physical activity intervention on schoolchildren's health-related quality of life: the active smarter kids (ASK) cluster-randomized controlled trial. Prev Med Rep. (2019) 13:1–4. 10.1016/j.pmedr.2018.11.002 30456052 PMC6234767

[36] Meyer U Schindler C Zahner L Ernst D Hebestreit H van Mechelen W. Long-term effect of a school-based physical activity program (KISS) on fitness and adiposity in children: a cluster-randomized controlled trial. PLoS ONE. (2014) 9:e87929. 10.1371/journal.pone.0087929 24498404 PMC3912178

[37] Omorou AY Langlois J Lecomte E Briancon S Vuillemin A. Cumulative and bidirectional association of physical activity and sedentary behaviour with health-related quality of life in adolescents. Qual Life Res. (2016) 25:1169–78. 10.1007/s11136-015-1172-7 26542533

[38] Gopinath B Baur LA Wang JJ Hardy LL Teber E Kifley A. Influence of physical activity and screen time on the retinal microvasculature in young children. Arterioscler Thromb Vasc Biol. (2011) 31:1233–9. 10.1161/ATVBAHA.110.219451 21508347

[39] Vella SA Cliff DP Magee CA Okely AD. Sports participation and parent-reported health-related quality of life in children: longitudinal associations. J Pediatr. (2014) 164:1469–74. 10.1016/j.jpeds.2014.01.071 24657117

[40] Gopinath B Hardy LL Baur LA Burlutsky G Mitchell P. Physical activity and sedentary behaviors and health-related quality of life in adolescents. Pediatrics. (2012) 130:e167-74. 10.1542/peds.2011-3637 22689863

[41] Wang H Sekine M Chen X Yamagami T Kagamimori S. Lifestyle at 3 years of age and quality of life (QOL) in first-year junior high school students in Japan: results of the toyama Birth cohort study. Qual Life Res. (2008) 17:257–65. 10.1007/s11136-007-9301-6 18157615

[42] Chen X Sekine M Hamanishi S Yamagami T Kagamimori S. Associations of lifestyle factors with quality of life (QOL) in Japanese children: a 3-year follow-up of the Toyama birth cohort study. Child Care Health Dev. (2005) 31:433–9. 10.1111/j.1365-2214.2005.00529.x 15948880

[43] Herman KM Hopman WM Craig CL. Are youth BMI and physical activity associated with better or worse than expected health-related quality of life in adulthood? The physical activity longitudinal study. Qual Life Res. (2010) 19:339–49. 10.1007/s11136-010-9586-8 20077141

[44] Rafferty R Breslin G Brennan D Hassan D A systematic review of school-based physical activity interventions on children's wellbeing. Int Rev Sport Exerc Psychol. (2016) 9:215–30. 10.1080/1750984X.2016.1164228

[45] Crews DJ Lochbaum MR Landers DM. Aerobic physical activity effects on psychological well-being in low-income hispanic children. Percept Mot Skills. (2004) 98:319–24. 10.2466/pms.98.1.319-324 15058892

[46] Van Hout R Young M Bassett S Hooft T Participation in sport and the perceptions of quality of life of high school learners in the Theewaterskloof municipality, South Africa. AJPHES. (2013) 19:612–22.

[47] Silva DAS Chaput JP Katzmarzyk PT Fogelholm M Hu G Maher C. Physical education classes, physical activity, and sedentary behavior in children. Med Sci Sports Exerc. (2018) 50:995–1004. 10.1249/MSS.0000000000001524 29252970

[48] Yap P Müller I Walter C Seelig H Gerber M Steinmann P. Disease, activity and schoolchildren's health (DASH) in Port Elizabeth, South Africa: a study protocol. BMC Public Health. (2015) 15:1285. 10.1186/s12889-015-2636-y 26700478 PMC4690231

[49] Filmer D Pritchett LH. Estimating wealth effects without expenditure data - or tears: an application to educational enrollments in states of India. Demography. (2001) 38:115–32. 10.1353/dem.2001.0003 11227840

[50] The Kidscreen Group Europe The KIDSCREEN Questionnaires: Quality of Life Questionnaires for Children and Adolescents (2006).

[51] Ravens-Sieberer U Auquier P Erhart M Gosch A Rajmil L Bruil J. The KIDSCREEN-27 quality of life measure for children and adolescents: psychometric results from a cross-cultural survey in 13 European countries. Qual Life Res. (2007) 16:1347–56. 10.1007/s11136-007-9240-2 17668292

[52] Ravens-Sieberer U Erhart M Rajmil L Herdman M Auquier P Bruil J. Reliability, construct and criterion validity of the KIDSCREEN-10 score: a short measure for children and adolescents' well-being and health-related quality of life. Qual Life Res. (2010) 19:1487–500. 10.1007/s11136-010-9706-5 20668950 PMC2977059

[53] Inchley J Currie D Young T Samdal O Torsheim T Auguston L Growing Up Unequal: Gender and Socioeconomic Differences in Young People's Health and Well-Being. Health Behaviour in School-Aged Children (HBSC) Study: International Report From the 2013/2014 Survey. Geneva: World Health Organization (2016).

[54] Prochaska JJ Sallis JF Long B. A physical activity screening measure for use with adolescents in primary care. Arch Pediatr Adolesc Med. (2001) 155:554–9. 10.1001/archpedi.155.5.554 11343497

[55] Galan I Boix R Medrano MJ Ramos P Rivera F Pastor-Barriuso R. Physical activity and self-reported health status among adolescents: a cross-sectional population-based study. BMJ Open. (2013) 3:e002644. 10.1136/bmjopen-2013-002644 23676798 PMC3657658

[56] Léger LA Mercier D Gadoury C Lambert J. The multistage 20 meter shuttle run test for aerobic fitness. J Sports Sci. (1988) 6:93–101. 10.1080/02640418808729800 3184250

[57] Mayorga-Vega D Aguilar-Soto P Viciana J. Criterion-related validity of the 20-m shuttle run test for estimating cardiorespiratory fitness: a meta-analysis. J Sports Sci Med. (2015) 14:536–47. 26336340 PMC4541117

[58] Lindwall M Lindgren EC The effects of a 6-month exercise intervention programme on physical self-perceptions and social physique anxiety in non-physically active adolescent Swedish girls. Psychol Sport Exerc. (2005) 6:643–58. 10.1016/j.psychsport.2005.03.003

[59] Ekeland E Heian F Hagen KB Can exercise improve self esteem in children and young people? A systematic review of randomised controlled trials. Br J Sports Med. (2005) 39:792–8. 10.1136/bjsm.2004.017707 16244186 PMC1725055

[60] Morales PF Sanchez-Lopez M Moya-Martinez P Garcia-Prieto JC Martinez-Andres M Garcia NL. Health-related quality of life, obesity, and fitness in schoolchildren: the Cuenca study. Qual Life Res. (2013) 22:1515–23. 10.1007/s11136-012-0282-8 23054495

[61] Myers J McAuley P Lavie CJ Despres JP Arena R Kokkinos P. Physical activity and cardiorespiratory fitness as major markers of cardiovascular risk: their independent and interwoven importance to health status. Prog Cardiovasc Dis. (2015) 57:306–14. 10.1016/j.pcad.2014.09.011 25269064

[62] Bouchard C Malina RM Perusse L Genetics of Fitness and Physical Activity. Champaign, IL: Human Kinetics (1997).

[63] Lindwall M Ljung T Hadzibajramovic E Jonsdottir I Self-reported physical activity and aerobic fitness are differently related to mental health. Ment Health Phys Act. (2012) 5:28–34. 10.1016/j.mhpa.2011.12.003

[64] Byrne BM Structural Equation Modeling with AMOS. Basic Concepts, Applications, and Programming. New York: Taylor & Francis (2010).

[65] Evaristo S Moreira C Lopes L Oliveira A Abreu S Agostinis-Sobrinho C. Muscular fitness and cardiorespiratory fitness are associated with health-related quality of life: results from labmed physical activity study. J Exerc Sci Fit. (2019) 17:55–61. 10.1016/j.jesf.2019.01.002 30740134 PMC6353732

[66] Andersen JR Natvig GK Aadland E Moe VF Kolotkin RL Anderssen SA. Associations between health-related quality of life, cardiorespiratory fitness, muscle strength, physical activity and waist circumference in 10-year-old children: the ASK study. Qual Life Res. (2017) 26:3421–8. 10.1007/s11136-017-1634-1 28656535

[67] Palacio-Vieira JA Villalonga-Olives E Valderas JM Espallargues M Herdman M Berra S. Changes in health-related quality of life (HRQoL) in a population-based sample of children and adolescents after 3 years of follow-up. Qual Life Res. (2008) 17:1207–15. 10.1007/s11136-008-9405-7 18931941

[68] Bisegger C Cloetta B von Rueden U Abel T Ravens-Sieberer U European Kidscreen G. Health-related quality of life: gender differences in childhood and adolescence. Soz Praventivmed. (2005) 50:281–91. 10.1007/s00038-005-4094-2 16300172

[69] Chen G Ratcliffe J Olds T Magarey A Jones M Leslie E. BMI, health behaviors, and quality of life in children and adolescents: a school-based study. Pediatrics. (2014) 133:e868-74. 10.1542/peds.2013-0622 24590749

[70] Griffiths LJ Parsons TJ Hill AJ. Self-esteem and quality of life in obese children and adolescents: a systematic review. Int J Pediatr Obes. (2010) 5:282–304. 10.3109/17477160903473697 20210677

[71] Armstrong MEG Lambert MI Sharwood KA Lambert EV. Obesity and overweight in South African primary school children—the Health of the Nation Study. JEMDSA. (2014) 11:52–63. 10.1080/22201009.2006.10872144 16751921

[72] von Rueden U Gosch A Rajmil L Bisegger C Ravens-Sieberer U. Socioeconomic determinants of health related quality of life in childhood and adolescence: results from a European study. J Epidemiol Community Health. (2006) 60:130–5. 10.1136/jech.2005.039792 16415261 PMC2566139

[73] Haugland S Wold B Torsheim T. Relieving the pressure? The role of physical activity in the relationship between school-related stress and adolescent health complaints. Res Q Exerc Sport. (2003) 74:127–35. 10.1080/02701367.2003.10609074 12848225

[74] Scott JJ Morgan PJ Plotnikoff RC Lubans DR. Reliability and validity of a single-item physical activity measure for adolescents. J Paediatr Child Health. (2015) 51:787–93. 10.1111/jpc.12836 25643749

[75] Geiser C Data Analysis with MPlus. New York, NY: Guilford Publications (2010). 10.1007/978-3-531-92042-9

